# The influence of metaboreflex activation on pulmonary pressure with combined chemoreflex activation in acute and chronic hypoxia

**DOI:** 10.1113/JP290512

**Published:** 2026-06-17

**Authors:** Lauren E. Maier, Elliott J. Jenkins, Andrew Douglas, Jack Talbot, Alexandra Williams, Jonathan Moore, Craig D. Steinback, Liam D. Corr, Zoe H. Adams, Fabio Giuseppe Laginestra, Markus Amann, Philip N. Ainslie, Travis D. Gibbons, Mike Stembridge

**Affiliations:** 1 Cardiff School of Sport and Health Sciences, Cardiff Metropolitan University, Cardiff, Wales, UK; 2 Centre for Heart, Lung and Vascular Health, School of Health and Exercise Sciences, University of British Columbia Okanagan, Kelowna, Canada; 3 Institute for Applied Human Physiology, Department of Sport and Exercise Sciences, School of Psychology and Sports Science, College of Medicine and Health, Bangor University, Bangor, Wales, UK; 4 Neurovascular Health Lab, Faculty of Kinesiology, Sport, and Recreation, University of Alberta, Edmonton, AB, Canada; 5 Department of Anaesthesiology, University of Utah, Salt Lake City, UT, USA; 6 Department of Biological Sciences, Northern Arizona University, Flagstaff, AZ, USA

**Keywords:** high altitude, hypoxia, hypoxic pulmonary vasoconstriction, metaboreflex, pulmonary artery

## Abstract

Sympathetic activation arising from both the peripheral chemoreflex in hypoxia and muscle metaboreflex during exercise mediate an increase in pulmonary artery systolic pressure (PASP). We tested the hypothesis that coactivation of the chemoreflex and metaboreflex would augment the PASP response, as has been shown previously for heart rate and ventilation. We conducted two experimental studies: (i) a laboratory study (*n* = 15) in normoxia and acute isocapnic hypoxia (45 mmHg $P_{{ETO}_{2}}$) and (ii) a field study (*n* = 14) at sea level (344 m) and after 4–6 days of acclimatisation to high altitude (3800 m). In both studies, participants performed 3 min of isometric handgrip at 30% of maximal voluntary contraction followed by 3 min of post-exercise circulatory occlusion (PECO). PASP was assessed via transthoracic echocardiography. In Study 1, isocapnic hypoxia reduced $P_{{ETO}_{2}}$ (44.3 ± 5.3 mHg; *P* < 0.001) but maintained $P_{{ETCO}_{2}}$ (36.8 ± 2.3; *P* = 0.931) compared to normoxia. Both PECO (+5.3 ± 5.3 mmHg; *P* = 0.044) and hypoxia (+4.0 ± 2.0 mmHg; *P* = 0.016) elevated PASP from baseline, whereas combined hypoxic PECO further increased PASP (+8.4 ± 3.7 mmHg, *P* = 0.009). These responses were unaltered following acclimatisation to 3800 m in Study 2. Importantly, irrespective of the duration of hypoxic exposure and thus the degree of carotid body stimulation, the sum of the individual responses to hypoxia and PECO was not different compared to the combined hypoxic PECO stimulus in either acute (*P* = 0.806) or chronic hypoxia (*P* = 0.896). Therefore, although both chemoreflex activation with hypoxia and metaboreflex activation with PECO increase PASP, their coactivation results in an additive, not synergistic, response.

## Graphical Abstract

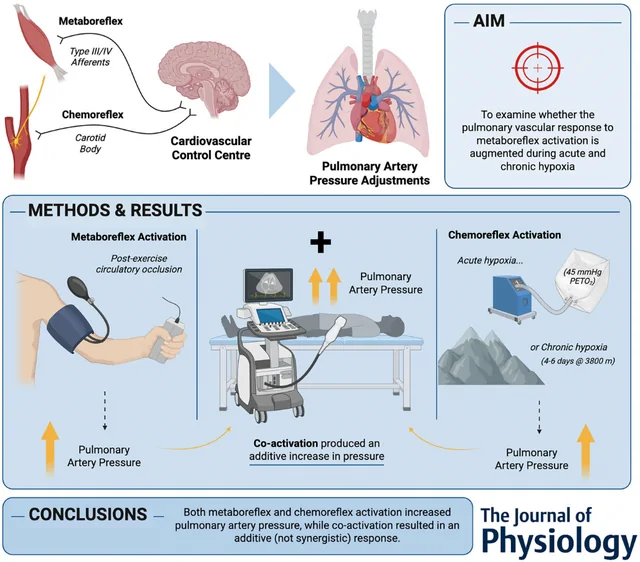

Activation of both the metaboreflex via post-exercise circulatory occlusion and the chemoreflex via acute and chronic hypoxia increased pulmonary artery systolic pressure. Coactivation of these reflexes further increased pulmonary artery pressure in an additive manner.

## Introduction

Cardiovascular and respiratory adjustments to exercise are primarily mediated via a combination of central command (Goodwin et al., 1972), baroreflex control (Bristow et al., 1971) and the muscle reflex (McCloskey & Mitchell, 1972). Neural feedback from the locomotor muscles is generated by mechanoreceptors and metaboreceptors, and communicated to the cardiovascular control centre via type III and IV afferents (Amann et al., 2015). This feedback leads to an appropriate increase in cardiac output, higher arterial pressure (Crisafulli et al., 2011; O’Leary, 1993; Sala-Mercado et al., 2006; Thurston et al., 2023), an increase in pulmonary ventilation (Amann et al., 2010) and elevated pulmonary artery pressure (Lykidis et al., 2008). Therefore, the muscle reflex plays a crucial role in ensuring ventilation–perfusion matching and oxygen delivery during exercise (Iannetta et al., 2024).

Exercise performed in hypoxia requires additional cardiorespiratory adjustments to ensure adequate gas exchange and oxygen delivery when oxygen availability is decreased. These are primarily mediated by the stimulation of oxygen-sensitive arterial chemoreceptors that increase sympathetic outflow, ventilation and mean arterial pressure (Blumberg et al., 1980; Gregor & Jänig, 1977; Powell et al., 1998). Hypoxia also increases discharge rates from group III/IV afferents, as demonstrated by perfusing resting muscle with hypoxic blood in anesthetised cats (Hill et al., 1992). Furthermore, although the chemoreflex and muscle reflex have been shown to independently influence cardiorespiratory control, their coactivation during exercise in acute hypoxia results in a hyper-additive increase in ventilation (Wan, Weavil, Thurston, Georgescu, Bledsoe et al., 2020), heart rate and systemic mean arterial pressure (Wan, Weavil, Thurston, Georgescu, Hureau et al., 2020). But whether this interactive effect is evident for the pulmonary vasculature, which itself is critical to regulating blood flow to ensure efficient gas exchange, remains to be explored.

The pulmonary vasculature is innervated by both sympathetic and parasympathetic fibres (Kummer, 2011). An increase in sympathetic activity, whether via acute hypoxia (Szidon & Flint, 1977) or the stimulation of the muscle metaboreflex (Lykidis et al., 2008), contributes to a rise in pulmonary artery pressure through contraction of the smooth muscle that surrounds the smaller pulmonary arterioles. During exercise in hypoxia, the increase in pulmonary pressure is greater for a given change in cardiac output compared to sea level (Groves et al., 1987), and the higher pulmonary pressure may limit exercise capacity (Naeije et al., 2010). Additional exercise-related reflex activation in hypoxia may be mediated by the metaboreflex, given that post-exercise circulatory occlusion (PECO) following handgrip in hypoxia markedly increased muscle sympathetic nerve activity and blood pressure compared to normoxic PECO (Houssiere et al., 2005). However, involvement of the chemoreflex is less certain because, unlike the systemic vasculature, the overriding effect of chemoreflex activation appears to be pulmonary vasodilatation mediated by parasympathetic activity. This is directly evidenced by a reduction in pulmonary pressure following denervation of the carotid bodies in animals (Levitzky et al., 1978; Naeije et al., 1989) and indirectly by the observation that a stronger acute hypoxic ventilatory response (i.e. higher chemoreflex sensitivity) is associated with a weaker pulmonary vasoconstrictive response (Albert & Swenson, 2014). It thus remains unclear whether chemoreflex activation modifies the metaboreflex effect on the pulmonary vasculature.

Hypoxic pulmonary vasoconstriction is not fully expressed for several days (Talbot et al., 2005). During this time, an increase in chemosensitivity also occurs via an increase in the number of type 1 glomus cells (Wang et al., 2008) and changes in ion channels, neurotransmitters and neuromodulators (Powell, 2007). The result is an increase in ventilation (Robbins, 2007); however, sympathetic responsiveness does not mirror changes in ventilation (Keir et al., 2019; Prasad et al., 2020), and the two appear to diverge during acclimatisation (Simpson et al., 2024). Although less established, there are data to suggest a similar sensitisation of type III/IV afferents. Dousset et al. (2001) observed an increased baseline group III/IV discharge and a greater response to stimulation after 45 days of chronic hypoxia compared to normoxia in rats (Amann & Kayser, 2009). Greater muscle afferent feedback and steeper pulmonary pressure–flow relationships have also been reported during pathological conditions such as heart failure (Lewis et al., 2013), where chemoreflex overactivity is common place (Kafi et al., 1998; Keir et al., 2020; Narkiewicz et al., 1999; Schultz & Sun, 2000) and sensitisation of the muscle pressor reflex is assumed to develop over time (Iannetta et al., 2025; Middlekauff et al., 2004; Piepoli et al., 2008; Scott et al., 2000). Therefore, the influence of chemoreflex and metaboreflex activation on the pulmonary vasculature may be modified with duration of exposure, but this is yet to be determined in healthy humans.

To address this, we explored the interaction between metaboreflex and chemoreflex activation during two experimental studies. First, we examined whether the pulmonary vascular response to metaboreflex activation via PECO was augmented during acute chemoreflex activation in isocapnic hypoxia. Second, we examined whether metaboreflex activation was augmented during prolonged poikilocapnic hypoxic exposure to high altitude at 3800 m. We hypothesised that (i) pulmonary pressure response to metaboreflex activation would be greater in acute isocapnic hypoxia *vs*. normoxia and (ii) acclimatisation to high altitude would further increase the pulmonary vascular response to metaboreflex.

## Methods

### Ethical approval

Two experimental studies were conducted: in a laboratory study conducted at sea level (32 m) where measures were taken during a normoxic normocapnic baseline and in acute isocapnic hypoxia (Study 1) and a in second field study where the same experimental measures were taken at baseline (344 m) and after 4–6 days at 3800 m above sea level (Study 2). The laboratory study was approved by the Cardiff School of Sport and Health Sciences Natural Research Ethics Committee (Sta-7417) and the University of Alberta Research Ethics Board (PRO00129389), whereas the field study was approved by the University of British Columbia Clinical Research Ethics Board (H22–01091). Written informed consent was obtained from all participants prior to participation and the study conformed to the *Declaration of Helsinki*, other than registration in a database.

### Experimental design

Two separate groups were recruited to take part in the two studies, with data collected in 15 participants (seven females) in acute hypoxia (Study 1) and in 14 participants (seven females) in chronic hypoxia (Study 2). Four participants were recruited for both studies, but the experiments were distinct and separated by >1 year. Participants were aged between 18 and 45 years, and were considered healthy (no history of cardiovascular, respiratory, nervous system, or metabolic disease). Participants visited the laboratory once in Study 1 and twice for Study 2 (once each at sea level and between 4 and 7 days of acclimatisation to high altitude). All participants abstained from exercise, alcohol and caffeine for 12 h prior to experimental visits. In Study 1, participants performed the protocol under two different conditions: normocapnic normoxia and isocapnic hypoxia (45 mmHg end-tidal oxygen). End-tidal partial pressures of oxygen ($P_{{ETO}_{2}}$) and carbon dioxide ($P_{{ETCO}_{2}}$) were manipulated using a dynamic end-tidal forcing system (AirForce; University of British Columbia, Canada) (Tymko et al., 2015), which delivers gas to an inspiratory reservoir through a mixing and humidification chamber. In the field study, $P_{{ETCO}_{2}}$ was maintained at high-altitude eupnoeic values through visual feedback for the participant and corresponding instruction to increase or decrease their ventilation. $P_{{ETCO}_{2}}$ was controlled in both studies as our intervention to activate the metaboreflex (details below) is known to stimulate ventilation (Braz et al., 2014), and hypocapnia has been shown to decrease pulmonary artery pressure (Balanos et al., 2003).

Following instrumentation, participants lay supine and performed three maximal voluntary contractions (MVCs) using a handgrip device (MLT003/D Grip Force Transducer; ADInstruments, Dunedin, New Zealand) to determine the exercise intensity. A 5 min baseline was collected at the start of each condition, allowing for stabilisation of end-tidal values. In Study 1, participants were exposed to either normoxia or isocapnic hypoxia via the end-tidal forcing system. In the last minute of baseline, participants remained in the supine position and resting echocardiographic images were obtained. Following baseline, participants performed 3 min of isometric handgrip exercise at 30% of their MVC. During the final minute of exercise, exercising echocardiographic images were taken. Moments (<3 s) prior to the end of exercise, a blood pressure cuff (pneumatic tourniquet E20 Rapid Cuff Inflator; Hokanson, Bellevue, WA, USA) was placed around the bicep of the exercising arm and inflated to a supra-systolic pressure (≥200 mmHg) to occlude blood flow and prolong metaboreflex activation. Participants then stopped exercising and the PECO lasted for 3 min. In the last minute of PECO, echocardiographic images were collected. Participants were given adequate rest (i.e. minimum 15 min) following the normoxia trial, preceding hypoxia, and it was confirmed that end-tidal partial pressures and cardiovascular responses had returned to baseline values prior to starting the next stage.

### Experimental measures

Upon arrival, basic anthropometric measurements were taken and then resting blood pressure, ECG and respiratory measurements were obtained. Measurements of heart rate and rhythm (ECG lead II), beat by beat blood pressure (NIBP system; ADInstruments), peripheral oxygen saturation ($S_{{pO}_{2}}$; AutoCorr 3304, Smiths Medical, Waukesha, WI, USA) and respiratory flow and end-tidal partial pressure of oxygen and carbon dixoide ($P_{{ETO}_{2}}$ and $P_{{ETCO}_{2}}$; Spirometer and Gas Analyzer; ADInstruments) were collected continuously at 1 kHz (Chart Pro, version 8.3.1; ADInstruments). All transthoracic echocardiography images were performed using a commercially available ultrasound machine (Study 1: Vivid S70; Study 2: Vivid IQ; GE Healthcare, Chalfont St Giles, Bucks, UK) with a 1.5–4 MHz-phased array transducer. All imaging was performed by experienced cardiac sonographers (MS, EJ and AW) in line with guidelines from the American Society of Echocardiography (Lang et al., 2005; Rudski et al., 2010). Images were taken from the parasternal short-axis and apical four-chamber views. Stroke volume (SV) was calculated from the cross-sectional area of the left ventricular outflow tract measured in the para-sternal long-axis view and the velocity-time integral of the left ventricular outflow tract (obtained using a pulsed wave Doppler in an apical five chamber view. SV was calculated as the product of the velocity-time integral and aortic area, and cardiac output (*Q*) was obtained by multiplication with heart rate as previously validated against thermodilution and direct Fick (Christie et al. 1987) and used extensively by our group under similar experimental conditions (Simpson et al., 2020; Stembridge et al., 2019). Pulmonary artery systolic pressure (PASP) was estimated using Bernoulli’s equation, based upon the measurement of the maximum velocity of the tricuspid regurgitation jet (Rudski et al., 2010).

### Statistical analysis

All statistical analyses were performed using Prism, version 10.4.1 (GraphPad Software Inc., San Diego, CA, USA). Results are reported as the mean ± SD. Differences in descriptive characteristics between low- and high-altitude were assessed using a paired *t* test. Two-way repeated measures ANOVA was used to assess differences between conditions (e.g. normoxia *vs*. acute hypoxia) and across the protocol (e.g. baseline, exercise, PECO). In the case of missing values, a linear mixed effects model was used. *Post hoc* comparisons were performed using the Holm–Sidak method where main effects were significant. Statistical significance was set *a priori P* < 0.05, and *P* values reported were corrected for multiple comparisons using the Holm–Sidak method.

## Results

Participant demographics for both Studies 1 and 2 are shown in Table 1.

### Study 1: acute hypoxia

#### Regulation of end-tidal gases and ventilatory responses.

Isocapnic hypoxia reduced resting $S_{{pO}_{2}}$ from 97.0 ± 1.1% to 87.2 ± 4.4% (*P* < 0.001). Baseline end-tidal gases were successfully maintained during handgrip exercise and PECO in normoxia (Table 2). By design, $P_{{ETO}_{2}}$ was lower during isocapnic hypoxia across baseline, exercise and PECO stages (Table 2). There was a main effect of exercise stage on $P_{{ETCO}_{2}}$ (*P* = 0.033) and *post hoc* comparisons found an 0.5 mmHg elevation during isocapnic hypoxic exercise compared to hypoxic PECO (*P* = 0.049) (Table 2). Minute ventilation (${\dot{V}}_{E}$) was not different between baseline and PECO in either normoxia or hypoxia (*P* = 0.674) but was elevated during exercise (*P* = 0.003) (Table 2). Isocapnic hypoxia increased ${\dot{V}}_{E}$ compared to normoxia (*P* < 0.001). ${\dot{V}}_{E}$ was further elevated during hypoxic exercise (*P* < 0.001) but was not different between baseline isocapnic hypoxia and combined hypoxic PECO (*P* = 0.721) (Table 2).

#### Cardiovascular and pulmonary vascular responses.

**Figure 1.**
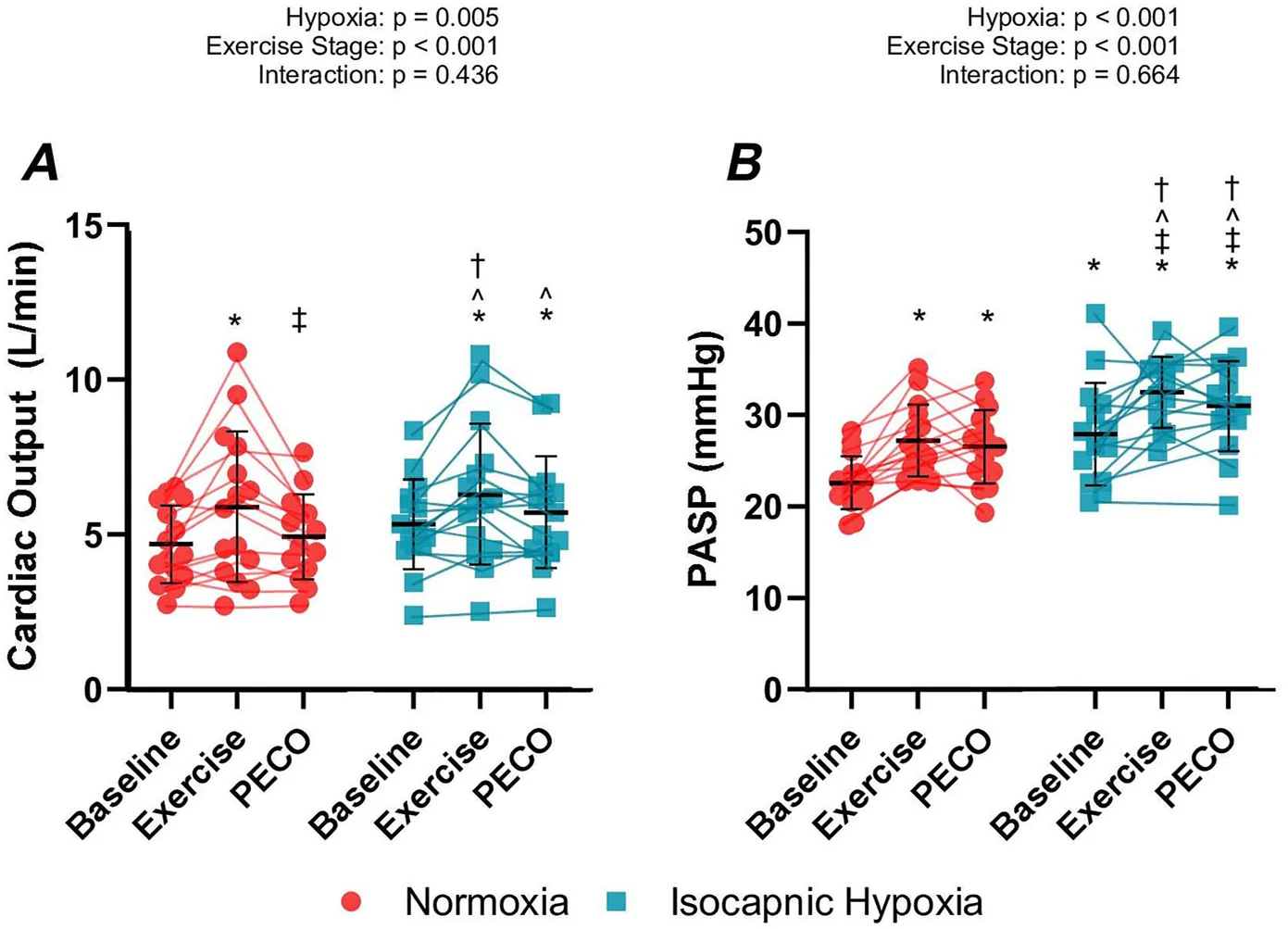
The effect of acute chemoreflex and metaboreflex activation on (A) cardiac ouput and (B) pulmonary artery systolic pressure *A*, cardiac output during baseline, exercise and post-exercise circulatory occlusion in normoxia and isocapnic hypoxia. *B*, pulmonary artery systolic pressure during baseline, exercise and post-exercise circulatory occlusion in normoxia and acute isocapnic hypoxia. Mixed model regressions were conducted to examine effects of isocapnic hypoxia and exercise stage on cardiac output and pulmonary artery systolic pressure. ∗*P* < 0.05 compared to normoxic baseline; ‡*P* < 0.05 compared to normoxic exercise; ˆ*P* < 0.05 compared to normoxic PECO; †*P* < 0.05 compared to isocapnic hypoxic baseline; *n* = 15 for all conditions except *Q* normoxia PECO, where *n* = 14 and PASP isocapnic hypoxic exercise, where *n* = 12. PASP, pulmonary artery systolic pressure; PECO, post-exercise circulatory occlusion.

Heart rate was increased during normoxic exercise and in all conditions under isocapnic hypoxia relative to normoxic baseline (Table 2). SV was not influenced by isocapnic hypoxia or exercise stage (i.e. exercise or PECO) (Table 2). There was a main effect of exercise stage on cardiac output, elevated with exercise in normoxia and hypoxia (*P* < 0.001). Cardiac output was also higher across exercise stages with isocapnic hypoxia (*P* = 0.005) (Fig. 1A). However, *post hoc* comparisons found that cardiac output was not elevated by PECO in normoxia or isocapnic hypoxia. There was a main effect of exercise stage on pulmonary vascular resistance (PVR) but no significant pairwise comparisons (Table 2).

PASP was significantly elevated from baseline (22.6 ± 2.9 mmHg) during normoxic exercise (27.2 ± 3.9 mmHg; *P* < 0.001) and PECO (26.5 ± 4.0 mmHg; *P* = 0.002) (Fig. 1B). PASP was also elevated at baseline in isocapnic hypoxia (27.9 ± 5.6 mmHg; *P* < 0.001) compared to normoxia and increased during hypoxic exercise (32.5 ± 3.9; *P* = 0.009) and PECO (31.0 ± 4,9 mmHg; *P* = 0.016) (Fig. 1B) from hypoxic baseline. However, there was no hypoxia × exercise stage interaction on PASP (*P* = 0.664).

**Figure 2.**
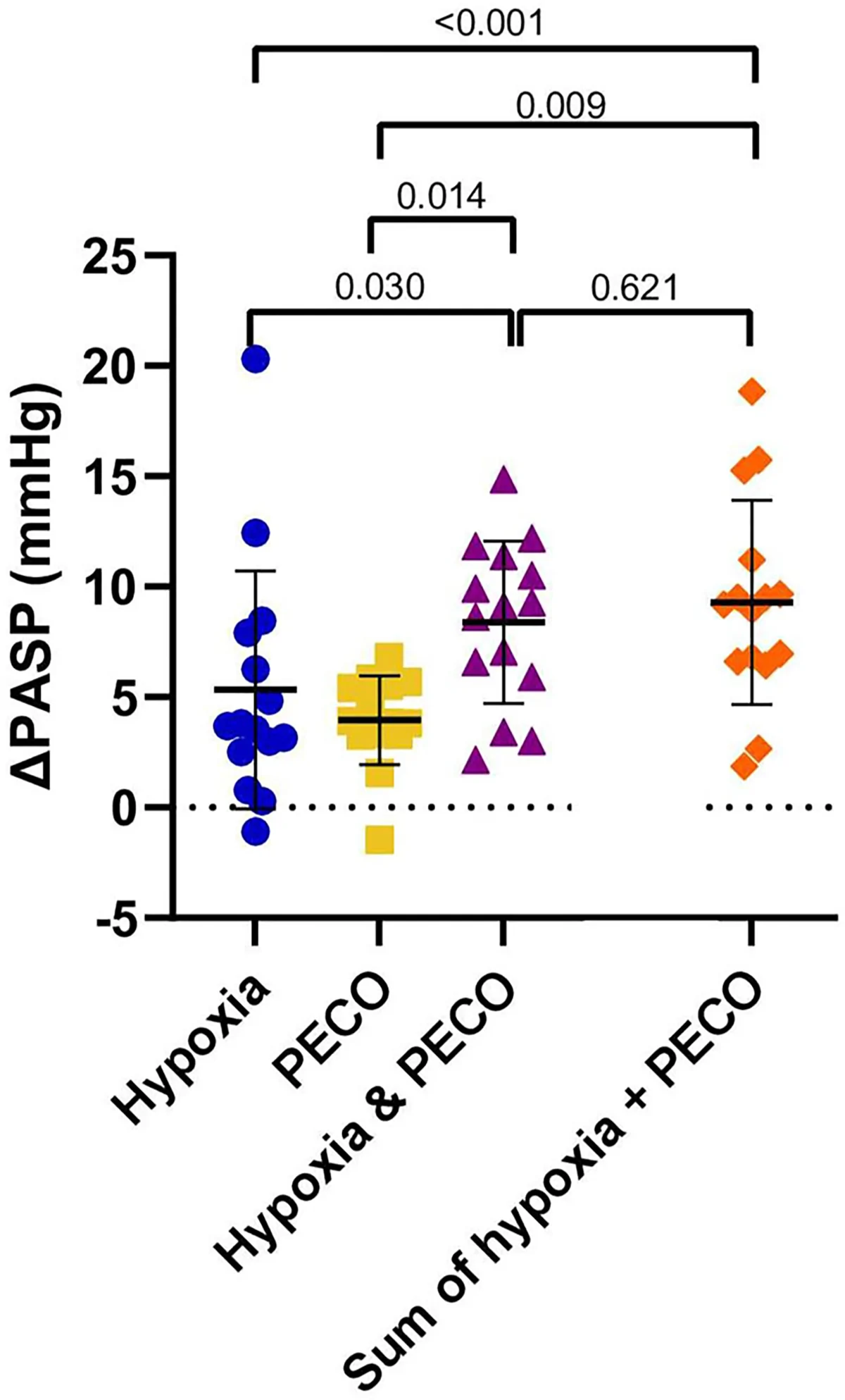
The change in pulmonary artery systolic pressure from normoxia with isocapnic hypoxia, post-exercise circulatory occlusion, combined isocapnic hypoxia and post-exercise circulatory occlusion, and the individual conditions added together One-way repeated measures ANOVA comparing the change in pulmonary artery systolic pressure identified a main effect of condition (*P* = 0.003). PASP, pulmonary artery systolic pressure; PECO, post-exercise circulatory occlusion.

When expressed as a delta *vs*. normoxic baseline (Fig. 2), the change in PASP was similar during hypoxia baseline and normoxic PECO. The combined stimulus (hypoxia and PECO) further increased PASP compared to hypoxia (*P* = 0.030) or PECO (*P* = 0.014) alone (Fig. 2). Importantly, however, the sum of the individual responses to hypoxia and PECO was not different compared to the combined isocapnic hypoxic PECO stimulus (*P* = 0.621) (Fig. 2).

#### Sex-based comparisons.

The change in PASP in females during isocapnic hypoxia (3.3 ± 2.3 mmHg), PECO (6.7 ± 7.4 mmHg) and combined isocapnic hypoxia and PECO (8.1 ± 4.4 mmHg) was similar compared to males during isocapnic hypoxia (4.5 ± 1.7 mmHg), PECO (4.2 ± 2.7 mmHg) and combined isocapnic hypoxia and PECO (8.6 ± 3.3 mmHg; main effect of sex, *P* = 0.839). Thus, participants were grouped together for analysis.

### Study 2: chronic altitude acclimatisation

#### Regulation of end-tidal gases and ventilatory responses.

High altitude exposure (4–6 days at 3800 m) reduced resting $S_{{pO}_{2}}$ from 98.4 ± 1.2% to 89.6 ± 2.0% (*P* < 0.001). $P_{{ETO}_{2}}$ was lower across exercise stages compared to sea level (*P* < 0.001) (Table 3). Resting $P_{{ETCO}_{2}}$ was higher at sea level compared with high altitude baseline (*P* < 0.001), but was effectively clamped during exercise (sea level, *P* = 0.531; high altitude, *P* = 0.111) and PECO (sea level, *P* = 0.993; high altitude, *P* > 0.999). ${\dot{V}}_{E}$ was different between altitudes (*P* < 0.001) and exercise stages (*P* < 0.001) (Table 3). However, pairwise comparisons found no significant difference between baseline and PECO at sea level (*P* = 0.259) or high altitude (*P* = 0.070).

#### Cardiovascular and pulmonary vascular responses.

**Figure 3.**
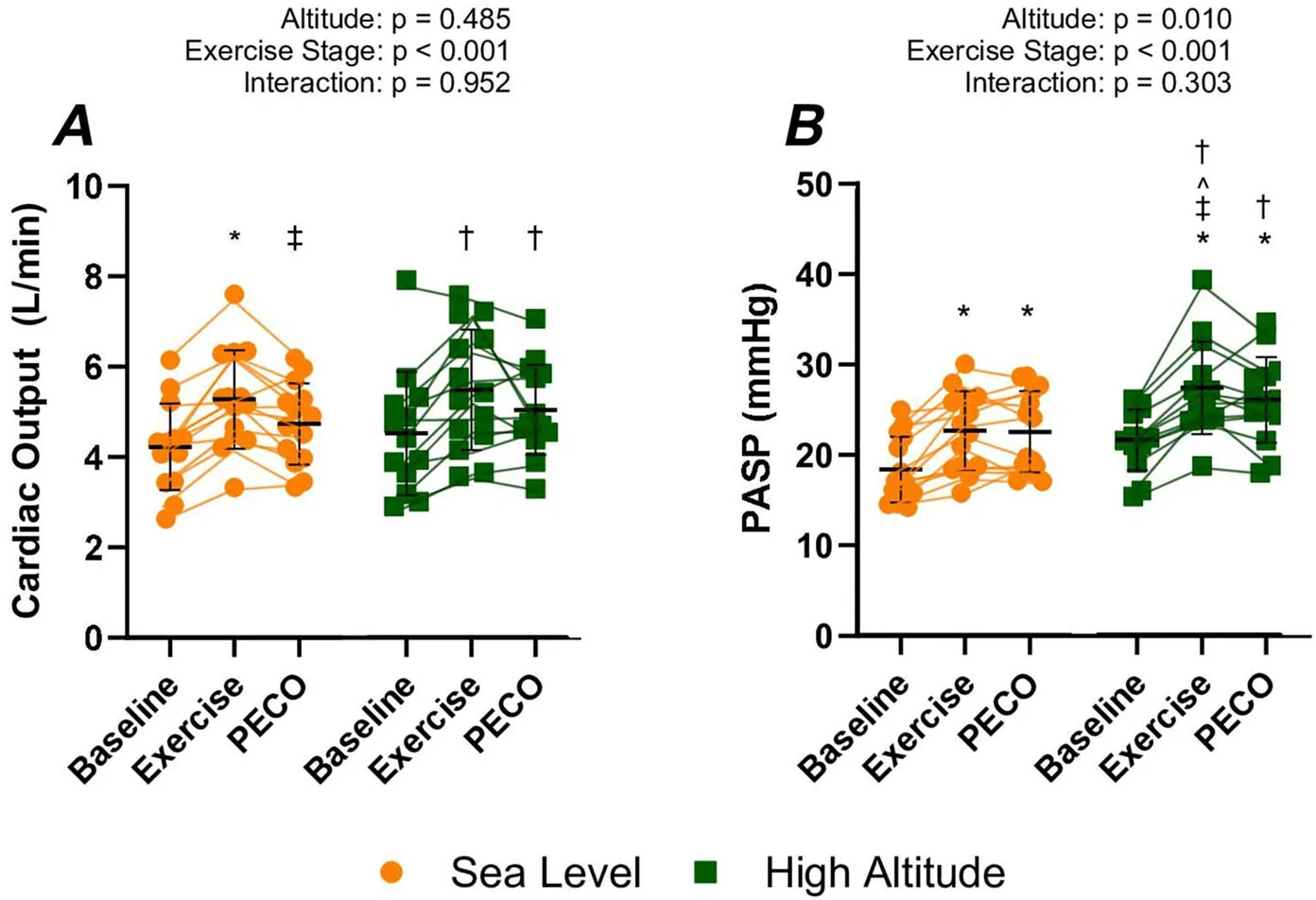
The effect of chronic chemoreflex and metaboreflex activation on *A* cardiac output and *B* pulmonary artery systolic pressure *A*, cardiac output during baseline, exercise, and post-exercise circulatory occlusion at sea level and high altitude. *B*, pulmonary artery systolic pressure during baseline, exercise and post-exercise circulatory occlusion at sea level and high altitude. Linear mixed model regressions were conducted comparing altitude and exercise stage for cardiac output and pulmonary artery systolic pressure. ∗*P* < 0.05 compared to sea level baseline; ‡*P* < 0.05 compared to sea level exercise; ˆ*P* < 0.05 compared to sea level PECO; †*P* < 0.05 compared to high altitude baseline; *n* = 14 for all conditions, except high altitude baseline, where *n* = 13. PASP, pulmonary artery systolic pressure; PECO, post-exercise circulatory occlusion.

Heart rate was elevated across all exercise stages at high altitude *vs*. sea level (*P* < 0.001) (Table 3). Pairwise comparisons identified an increase in heart rate at high altitude both at rest (*P* = 0.006) and during PECO (*P* < 0.001) compared to sea level. Exercise stage elevated heart rate, but individual comparisons found heart rate was not different from rest during PECO at sea level or high altitude (Table 3). SV was not different with altitude exposure (*P* = 0.134) or exercise stage (*P* = 0.184) (Table 3). There was no effect of altitude on cardiac output (*P* = 0.485), but exercise stage did have a main effect (*P* < 0.001) (Fig. 3A). *Post hoc* comparisons identified an increase in cardiac output from sea level baseline with exercise (*P* < 0.001) and PECO, although this failed to reach statistical significance for the latter (*P* = 0.053). Cardiac output during sea level exercise was higher than PECO (*P* = 0.038). At high altitude, both exercise and PECO significantly elevated cardiac output from baseline (*P* < 0.001 and *P* = 0.035, respectively). Cardiac output was not different between exercise and PECO at altitude (*P* = 0.110) (Fig. 3A). PVR was not different with altitude or exercise stage (Table 3).

There was a main effect of altitude (*P* = 0.010) and exercise stage (*P* < 0.001) on PASP, but no interaction (*P* = 0.303) (Fig. 3B). Exercise elevated PASP from baseline at sea level (18.4 ± 3.6 mmHg *vs*. 22.7 ± 4.4 mmHg, *P* < 0001) and high altitude (21.7 ± 3.4 mmHg *vs*. 27.4 ± 5.1 mmHg, *P* < 0.001). PASP remained similarly elevated during PECO at sea level (22.6 ± 4.5 mmHg, *P* < 0.001) and high altitude (26.1 ± 4.7 mmHg, *P* < 0.001) (Fig. 3B).

**Figure 4.**
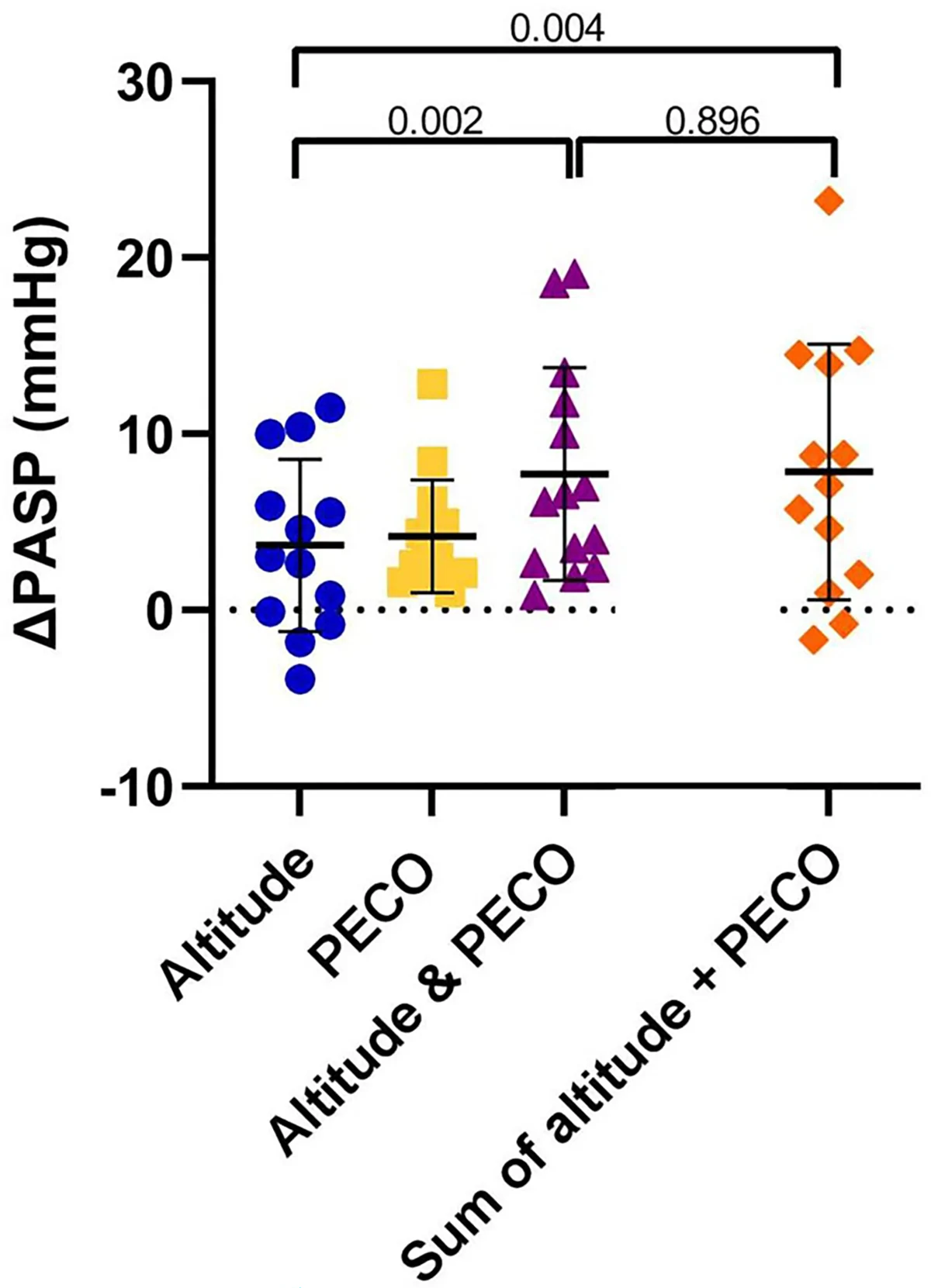
The change in pulmonary artery systolic pressure with post-exercise circulatory occlusion, altitude, combined post-exercise circulatory occlusion and altitude, and the individual conditions added together Mixed model analysis of the change in pulmonary artery systolic pressure identified a difference between conditions (*P* = 0.001). PASP, pulmonary artery systolic pressure; PECO, post-exercise circulatory occlusion.

When expressed as a delta *vs*. sea level baseline, there was a similar increase in PASP with altitude and PECO (*P* = 0.896), but the combined stimulus of PECO at altitude increased PASP greater than altitude alone (*P* = 0.002). However, PECO and altitude was not greater than PECO alone (*P* = 0.074). Similar to our results during acute isocapnic hypoxia in Study 1, the sum of altitude and PECO was not different compared to the combined altitude and PECO stimulus (*P* = 0.896) (Fig. 4).

#### Sex-based comparisons.

The change in PASP in females with high altitude (3.3 ± 4.8 mmHg), PECO (3.3 ± 2.5 mmHg) and combined high altitude and PECO (6.7 ± 6.5 mmHg) was similar compared to males at high altitude (4.1 ± 5.5 mmHg), PECO (5.0 ± 3.8 mmHg) and combined high altitude and PECO (9.2 ± 8.4 mmHg; main effect of sex, *P* = 0.901). Thus, participants were grouped together for analysis.

### Acute (Study 1) *vs*. chronic (Study 2) hypoxia

**Figure 5.**
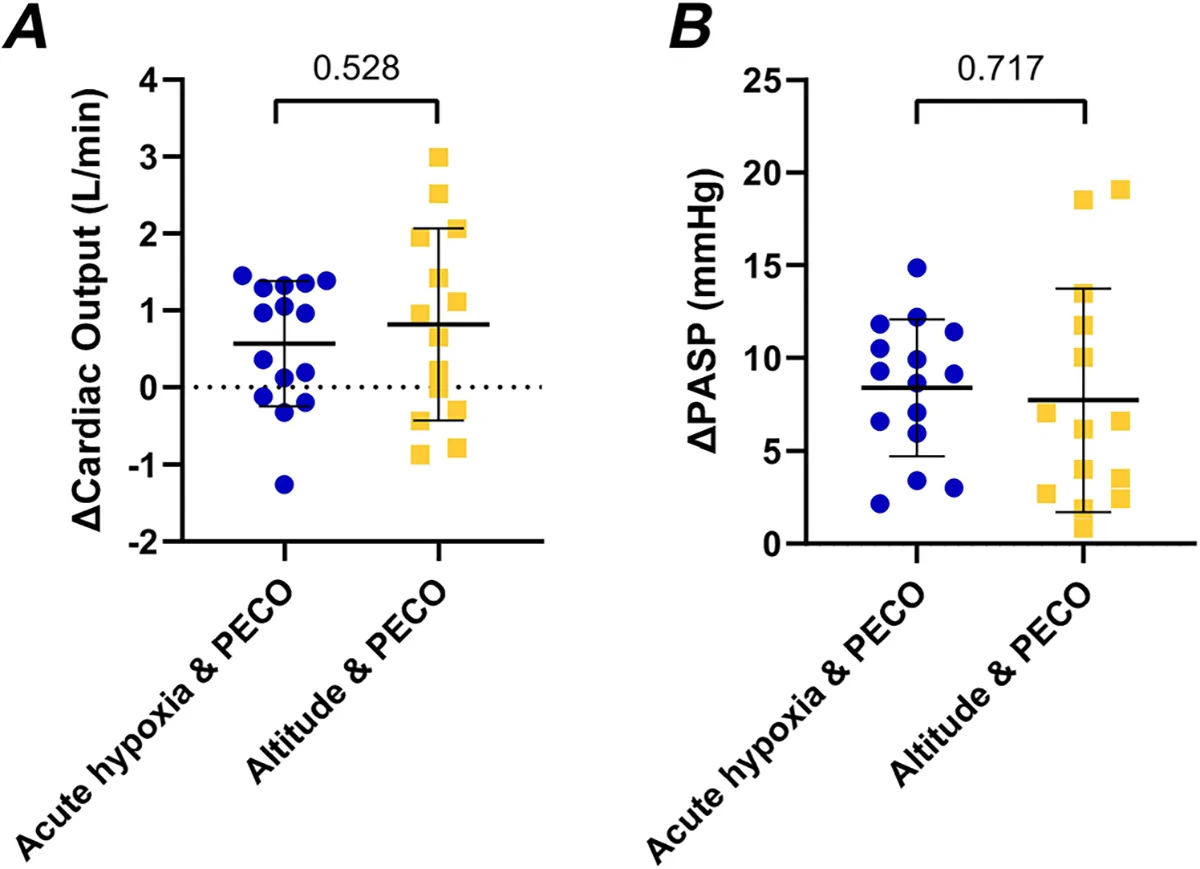
Comparing acute vs. chronic chemoreflex activation with combined metaboreflex activation on the *A* cardiac output and *B* pulmonary artery systolic pressure responses *A*, change in cardiac output from baseline to acute isocapnic hypoxic post-exercise circulatory occlusion compared to the change from baseline to post-exercise circulatory occlusion at altitude. *B*, change in pulmonary artery systolic pressure from baseline to acute isocapnic hypoxic post-exercise circulatory occlusion compared to the change from baseline to post-exercise circulatory occlusion at altitude. Conditions were compared with unpaired *t* tests; *n* = 15 for acute hypoxia conditions and *n* = 14 for altitude conditions. PASP, pulmonary artery systolic pressure; PECO, post-exercise circulatory occlusion.

With acclimatisation, resting ${\dot{V}}_{E}$ was reduced relative to acute hypoxia at sea level (*P* = 0.005). Resting HR was not different in acute *vs*. chronic hypoxia (*P* = 0.375). To test whether acclimatisation further augmented the pulmonary vascular response, the magnitude of the response to PECO in acute and chronic hypoxia was compared. As a result, no difference in the change in cardiac output (*P* = 0.528) or PASP (*P* = 0.717) was observed between the two time-domains of hypoxia (Fig. 5).

## Discussion

The present study aimed to determine whether activation of the metaboreflex during peripheral chemoreceptor activation leads to an augmented pulmonary artery pressure response. The primary findings were that: (i) metaboreflex and chemoreflex activation in isolation increased PASP and (ii) the absolute pulmonary artery pressure achieved during metaboreflex activation in acute and chronic hypoxia was greater than in normoxia; however, (iii) the increase in PASP during combined metaboreflex and chemoreflex activation was similar to the sum of the individual responses. Collectively, our data support an additive response of metaboreflex and chemoreflex activation via PECO and hypoxic exposure on pulmonary pressure.

### Influence of metaboreflex activation on PASP

Lykidis et al. (2008) were the first to report an increase in pulmonary artery pressure during metaboreflex activation in humans. In the present study, we confirm and extend these findings by demonstrating an increase in PASP during PECO in normoxia and acute and chronic hypoxia. An important distinction between our study and the work of Lykidis et al. (2008) is the control end-tidal gases during the PECO. PECO can augment ventilation (Lam et al., 2019; Plunkett et al., 2024) irrespective of hypoxia, as a result of the stimulatory influence of group III and IV skeletal muscle afferents on the respiratory drive during exercise (Amann et al., 2010), although this is not always the case (Bruce & White, 2015; Rowell et al., 1976). Increased ventilation would reduce PETCO_2_, which turn could attenuate the rise in PASP (Balanos et al., 2003), a phenomenon that has been observed in the cerebral circulation where hypocapnia resulted in greater vascular resistance during PECO (Braz et al., 2014). Therefore, we might have expected a larger change in PASP in our study, given that we clamped $P_{{ETCO}_{2}}$ to baseline values using either dynamic end-tidal forcing (Study 1) or paced ventilation (Study 2). However, we report a similar absolute change in PASP compared to Lykidis et al. (2008), potentially because of the slower kinetics of vasoactive response of the pulmonary artery to changes in carbon dioxide relative to the cerebral circulation (Balanos et al., 2003).

### Combined metaboreflex and chemoreflex activation

Previous work has demonstrated a hyper-additive interaction between chemoreceptor activation and II/IV muscle afferent activation, with the combined influence of hypoxia and II/IV muscle afferent feedback on ventilation (Wan, Weavil, Thurston, Georgescu, Bledsoe et al., 2020; Wan, Weavil, Thurston, Georgescu, Hureau et al., 2020), mean arterial pressure and heart rate (Wan, Weavil, Thurston, Georgescu, Hureau et al., 2020) to be greater than the sum of each reflex in isolation. Both studies used a sophisticated experimental model employing pharmacological blockade of lower limb muscle afferents (both metaboreceptors and mechanoreceptors) during single-leg voluntary knee extensions. By contrast to this work, we did not observe the same hyperadditive effect in the PASP response to combined activation. This may be explained by the experimental model because a number of other studies using post-exercise occlusion to stimulate metaboreceptors have consistently reported the sum of cardiorespiratory responses to hypoxia and exercise to be equal to the combined stressors (Delliaux et al., 2015; Edgell & Stickland, 2014; Lykidis et al., 2008; Seals et al., 1991; Silva et al., 2018). Despite the limitations of PECO (discussed below in our ‘Limitations’ section), others have still been able to demonstrate hyperadditive effects during handgrip exercise for example, potentiation of muscle sympathetic nerve activity in hypoxia *vs*. normoxia (Seals et al., 1991). By contrast, however, the absolute increase in PASP from rest to exercise in the current study was comparable in normoxia *vs*. hypoxia indicating an additive but not synergistic interaction of the chemoreflex and metaboreflex on the pulmonary vasculature. It is possible the higher intensity, dynamic exercise used by Seals et al. (1991) contributed to the observed potentiation; indeed, when ischemic isometric exercise was employed, the sympathetic responses were similar under normoxia and hypoxia. Thus, the hyper-additive interaction of the chemoreflex and metaboreflex may only occur above a certain exercise intensity.

### Consequences of prolonged hypoxia for pulmonary pressure response to metaboreflex activation

The effects of hypoxia on both the chemoreflex and pulmonary vasculature are time dependent. Hypoxic pulmonary vasoconstriction is fully expressed after ~8 h of high altitude exposure (Talbot et al., 2005); PASP then remains relatively stable during acclimatisation (Dorrington et al., 1997). In comparison, ventilatory chemosensitivity increases over the first 24–48 h (Dempsey et al., 2014; Robbins, 2007). Although chemoreflex activation can have differential effects on ventilation and sympathetic outflow (Fisher et al., 2018; Keir et al., 2019; Prasad et al., 2020), silencing the carotid body by using hyperoxia still lowers muscle sympathetic nerve activity following acclimatisation (Hansen & Sander, 2003). Therefore, we hypothesised that acclimatisation to high altitude would further enhance the pulmonary vascular response to metaboreflex. By contrast to our hypothesis, there were no differences in the effect of PECO on PASP between acute and chronic hypoxia. However, given we did not observe an interaction effect between the metaboreflex and chemoreflex at either altitude, this is not unexpected. Increased chemoreflex sensitivity with acclimatisation could not further augment the metaboreflex response when we found no evidence of an interaction between the reflexes in the acute setting.

### Pulmonary pressure and cardiovascular control during exercise

In our experiment, we show that isolated activation of the metaboreflex results in an increased pulmonary pressure, that is, greater metabolic stress at the muscle leads to higher afterload on the right heart. In an exercise setting, this would be counterproductive, as the right heart and pulmonary circulation needs to accommodate the entirety of the cardiac output whilst having a limited capacity to lower vascular resistance through recruitment and distension (Naeije & Chesler, 2012). Indeed, pulmonary vascular distensibility predicts maximal aerobic capacity in healthy individuals (Lalande et al., 2012), and the drop in pulmonary vascular resistance expected during exercise is attenuated with ageing and cardiovascular disease progression (Butler et al., 1999; Kovacs et al., 2012). Moreover, we have recently shown that the increase in pulmonary artery pressure seen with exercise drives sympathetic activity directed towards the skeletal muscle vasculature during moderate-intensity cycling (Ewalts et al., 2025). Exercise-induced sympathetic activation is derived from mechanoreceptors located in the pulmonary artery and bifurcation (Moore & Stembridge, 2025; Moore et al., 2004, 2011), which are increasingly recognised as important modulators of sympathetic vasoconstrictor outflow (Plunkett Sayegh et al., 2025). Logically, pulmonary pressure cannot be a stimulus for sympathetic activation to the systemic vasculature at the same time as being sensitive to increase in sympathetic outflow originating from the muscle. Hence, the modulating influence of skeletal muscle mechanoreceptors must also be considered. Our model of PECO only activates metaboreceptors, but, during dynamic exercise, muscle mechanoreceptors would also be activated. White et al. (2013) conducted measurements of pulmonary artery pressure and cardiac output during PECO (to activate metaboreceptors) followed by combined PECO with passive calf stretch (to simultaneously activate both metabo- and mechanoreceptors). As per their previous work investigating metaboreflex in isolation, White et al. (2013) reported an increase in pulmonary artery pressure during PECO, whereas cardiac output returned to baseline, indicating pulmonary vasoconstriction. When passive stretch was added to PECO, pulmonary pressure remained similar to the PECO condition, but cardiac output increased significantly. Given an increase in flow would be expected to increase pressure (Balanos et al., 2005), no change in pulmonary pressure can be interpreted as a decrease in pulmonary vascular resistance, probably mediated by parasympathetic withdrawal (Fisher et al., 2010; Gladwell & Coote, 2002). Although it may seem counterintuitive for parasympathetic withdrawal to lower vascular resistance, vagal blockade via atropine causes pulmonary vasodilatation when vascular tone is already elevated by sympathetic activation (Vanhoutte, 1974; Wilson et al., 1995). Therefore, coactivation of metaboreceptors and mechanoreceptors probably prevents a significant rise in pulmonary artery pressure during exercise that would otherwise limit exercise capacity as a result of an increased right ventricular load and systemic vasoconstriction.

### Potential influence of sex

Finally, it is important to consider the role of sex in pulmonary vascular responses to chemo- and metaboreflex activation. Females may have a blunted early acclimatory response of pulmonary arterial systolic pressure to isocapnic hypoxia (Fatemian et al., 2016) and cardiac responses may differ between sexes during concomitant chemo- and metaboreceptor activation (Boulet et al., 2022). However, *post hoc* analyses did not identify an effect of sex on our *a priori* outcome; thus, sex does not appear to influence our interpretation.

### Perspectives

Historically, the pulmonary vasculature was primarily considered to be influenced passively by cardiac output, intrathoracic pressure, respiratory gases and local vasoactive processes (Naeije & Chesler, 2012; Plunkett, Paton et al., 2025; Swenson, 2013). More recently, there has been renewed interest in neural control of the pulmonary circulation and therapeutic targeting of these pathways in pulmonary hypertension (Zhang et al., 2022). Clinical conditions such as pulmonary arterial hypertension and heart failure are often characterised by higher sympathetic activity at rest, and sympathetic hyperactivity during these conditions is related to higher mortality (Grassi et al., 2019; Velez-Roa et al., 2004). The chemoreflex has been shown to contribute to these tonic elevations in sympathetic activity (Schultz et al., 2015; Velez-Roa et al., 2004). Moreover, the muscle metaboreflex response to exercise is exaggerated in heart failure (Amann et al., 2014; Piepoli et al., 1996), with a steeper relationship between pulmonary pressure and cardiac output evident in pulmonary hypertension and heart failure patients (Lewis et al., 2013). Therefore, understanding the interaction between different components of reflex control of the pulmonary circulation is key to addressing a hallmark of cardiovascular disease that negatively influences quality of life through exercise limitation (Haarmann et al., 2016), and is predictive of all-cause mortality (Barretto et al., 2009) and sudden cardiac death (Brouwer et al., 1996). Whether the additive response we observed in healthy participants is mirrored in clinical conditions characterised by increased sensitivity of the chemoreflex and metaboreflex remains to be explored.

### Limitations

We utilised forearm isometric exercise with subsequent occlusion to stimulate the muscle metaboreflex, which carries several limitations that should be acknowledged. First, autonomic activity differs during occlusion at rest as a result of of the absence of mechanoreceptor activation and central command that will influence autonomic balance, as discussed above. However, the model used in the present study allows the investigation of the specific action of muscle metaboreceptors in hypoxia, albeit within the specific context under study, with caution needed when generalising to whole body exercise. Second, occlusion stimulates a subset of metaboreceptors that are not active during conventional exercise (Jankowski et al., 2013; Pollak et al., 2014). However, this model of metaboreceptor activation has still been able to detect potentiation of sympathetic tone during the exercise bout that precedes the occlusion (Seals et al., 1991). We also utilised an indirect method to measure pulmonary pressure. Invasive right heart catheterisation is the gold standard for the assessment of pulmonary haemodynamics; however, echocardiography is a recognised technique for the assessment of right heart function (Rudski et al., 2010) that correlates well with invasive methodologies (Currie et al., 1985; Yock & Popp, 1984). Lastly, the *a priori* question of the present study was to determine whether there was a hyper-additive effect of the metabo- and chemoreflexes on PASP; thus, we may not have been powered to detect sex-based differences. Furthermore, we were unable to control for menstrual cycle phase or contraceptive use in Study 2 as a result of the nature of research expeditions, and chose not to control for these in Study 1 because it was not the *a priori* research question. As a result, the role of sex in these outcomes is further confounded by variable hormone concentrations.

## Conclusions

The present study aimed to determine whether peripheral chemoreceptor activation with added metaboreflex stimulation results in an augmented pulmonary artery pressure response. We found that metaboreflex activation with PECO and chemoreflex activation via arterial hypoxemia both separately stimulate increases in PASP. When combined, there is an additive interaction that further increases PASP in healthy individuals.

## Supplementary Material

Supplementary mat

Supporting information

Additional supporting information can be found online in the Supporting Information section at the end of the HTML view of the article. Supporting information files available:

Peer Review History

The peer review history is available in the Supporting Information section of this article (https://doi.org/10.1113/JP290512#support-information-section).

## Figures and Tables

**Table 1.** Participant characteristics

	Study 1: acute hypoxia	Study 2: chronic hypoxia

*n* (male/female)	15 (8/7)	14 (7/7)
Age (years)	27 ± 5	29 ± 4
Height (cm)	174.0 ± 8.3	172.9 ± 5.7
Mass (kg)	74.6 ± 14.1	65.7 ± 7.9

**Table 2.** Cardio- and pulmonary vascular outcomes during baseline, exercise, and PECO in normoxia and acute isocapnic hypoxia

Normoxia							Isocapnic hypoxia		
	
	Baseline	Exercise	PECO	Baseline	Exercise	PECO	Hypoxia main effect	Exercise stage main effect	Interaction

HR (beats min^−1^)	61.8 ± 11.0	78.3 ± 17.2*	66.5 ± 12.1^‡^	69.1 ± 13.7*	83.7 ± 19.3^*,‡,^,†^	73.3 ± 14.7^*,†,^,§^	**0.004**	**<0.001**	0.772
MAP (mmHg)	85.9 ± 6.6	100.2 ± 8.6*	99.5 ± 6.9*	88.8 ± 9.1^*,‡,^^	104.5 ± 10.2^*,‡,^,†^	96.6 ± 8.9^*,‡,^,†,§^	0.521	**<0.001**	**<0.001**
$P_{{ETO}_{2}}$ (mmHg)	92.1 ± 7.1	91.7 ± 8.2	93.0 ± 8.6	44.3 ± 5.3*	44.4 ± 5.3^†^	44.7 ± 5.5^^^	**<0.001**	0.708	0.855
$P_{{ETCO}_{2}}$ (mmHg)	36.9 ± 2.4	37.0 ± 2.5	36.6 ± 2.0	36.8 ± 2.3	37.1 ± 2.7	36.6 ± 2.5^§^	0.931	0.033	0.635
${\dot{V}}_{E}$ (L min^−1^)	14.5 ± 3.2	17.4 ± 4.6*	15.1 ± 4.8^‡^	20.4 ± 6.7^*,‡,^^	26.5 ± 8.3^*,‡,^,†^	19.8 ± 6.2^*,‡,^,§^	**<0.001**	**<0.001**	**<0.001**
Stroke volume (mL)	76.5 ± 17.4	73.7 ± 18.8	74.5 ± 18.4 (14)	79.6 ± 20.2	75.4 ± 17.2	79.5 ± 21.4	0.072	0.137	0.681
PVR (mmHg L^−1^ min^−1^)	5.1 ± 1.4	5.3 ± 1.9	5.7 ± 1.5 (14)	5.6 ± 1.7	5.7 ± 2.1 (13)	5.9 ± 1.7	0.295	**0.039**	0.626

* *P* < 0.05 compared to normoxic baseline.

‡ *P* < 0.05 compared to normoxic exercise.

^ *P* < 0.05 compared to normoxic PECO.

† *P* < 0.05 compared to isocapnic hypoxia baseline.

§ *P* < 0.05 compared to isocapnic hypoxic exercise.

*n* = 15 unless specified in parentheses. Significant *P-values* bolded.

Abbreviations: HR, heart rate; MAP, mean arterial pressure; PECO, post-exercise circulatory occlusion; PVR, pulmonary vascular resistance.

**Table 3.** Cardio- and pulmonary vascular outcomes during baseline, exercise, and PECO at sea level and high altitude (3800 m)

Sea level				High altitude					
	Baseline	Exercise	PECO	Baseline	Exercise	PECO	Altitude main effect	Exercise stage main effect	Interaction

HR (beats min^−1^)	61.9 ± 9.5	73.3 ± 9.5*	59.3 ± 19.0^‡^	73.7 ± 13.3^*,^^	83.4 ± 11.8^*,‡,^,†^	74.1 ± 13.4^*,^,§^	**<0.001**	**<0.001**	0.545
MAP (mmHg)	75.0 ± 10.0	89.9 ± 11.1*	86.8 ± 12.4 (13)*	88.5 ± 10.1*	104.5 ± 12.8^*,‡,^,†^	100.6 ± 12.0*,†	**0.001**	**<0.001**	0.861
$P_{{ETO}_{2}}$ (mmHg)	93.8 ± 8.2	96.2 ± 8.2*	94.6 ± 7.8	52.7 ± 2.9 (13)^*,‡,^^	“ 54.8 ± 2.8^*,‡,^^	52.7 ± 3.1^*,‡,^^	**<0.001**	**0.004**	0.692
$P_{{ETCO}_{2}}$ (mmHg)	36.9 ± 4.7	36.4 ± 5.0	36.7 ± 4.7	29.6 ± 1.7^*,‡,^^	28.9 ± 1.8^*,‡,^^	29.7 ± 2.1 (13)^*,‡,^^	**<0.001**	**0.019**	0.5539
${\dot{V}}_{E}$ (L min^−1^)	11.2 ± 3.6	13.8 ± 3.1*	10.3 ± 2.8^‡^	14.2 ± 3.7^*,^^	17.4 ± 4.1^*,‡,^,†^	12.8 ± 3.1^^,§^	**<0.001**	**<0.001**	0.430
Stroke volume (mL)	76.6 ± 18.8	75.1 ± 16.0	78.6 ± 18.0	68.2 ± 17.4 (13)	68.6 ± 13.3	72.3 ± 18.6	0.134	0.184	0.825
PVR (mmHg L^−1^ min^−1^)	4.5 ± 1.2	4.5 ± 1.2	4.9 ± 1.2	5.2 ± 1.4 (12)	5.2 ± 1.1	5.3 ± 1.3	0.140	0.175	0.683

* *P* < 0.05 compared to sea level baseline.

‡ *P* < 0.05 compared to sea level exercise.

^ *P* < 0.05 compared to sea level PECO.

† *P* < 0.05 compared to high altitude baseline.

§ *P* < 0.05 compared to high altitude exercise.

*n* = 14 unless specified in parentheses. All significant *P-values* bolded.

Abbreviations: HR, heart rate; MAP, mean arterial pressure; PECO, post-exercise circulatory occlusion; PVR, pulmonary vascular resistance.

## Data Availability

Data are available from the corresponding author upon reasonable request.

## Key points

- Activation of the peripheral chemoreflex (e.g. hypoxia) and muscle metaboreflex (e.g. exercise) both increase heart rate, arterial pressure, ventilation and pulmonary pressure.
- There is evidence of an augmented muscle metaboreflex response for heart rate and ventilation in acute hypoxia, but whether the same effect is seen in the pulmonary vasculature has not been explored.
- We completed two experimental studies where pulmonary artery systolic pressure (PASP) was assessed during post-exercise circulatory occlusion (PECO) in response to acute isocapnic hypoxia and following acclimatisation to high altitude hypoxia (3800 m).
- Independent activation of the chemoreflex at rest and metaboreflex during PECO elevated PASP in both acute and chronic hypoxia.
- When combined, metaboreflex activation with PECO and chemoreflex activation with hypoxia have an additive but not interactive effect on PASP, irrespective of the duration of hypoxic exposure.
