# Supplementary material for: The influence of metaboreflex activation on pulmonary pressure with combined chemoreflex activation in acute and chronic hypoxia
Source: J Physiol. Author manuscript; Available in PMC 2026 Sep 18. (PMC13587138; doi:10.1113/JP290512)
Supplement: Supplementary file 1 [file NIHMS2205801-supplement-Supplementary_mat.pdf]

The Journal of Physiology

The influence of metaboreflex activation on pulmonary pressure with combined chemoreflex activation in acute and chronic hypoxia

Lauren E Maier, Elliott J Jenkins, Andrew JM Douglas, Jack S Talbot, Alexandra Mackenzie Williams, Jonathan P Moore, Craig Steinback, Liam Corr, Zoe H. Adams, Fabio Giuseppe Laginestra, Markus Amann, Philip N Ainslie, Travis D Gibbons, and Mike Stembridge
DOI: 10.1113/JP290512

Corresponding author(s): Mike Stembridge (mstembridge@cardiffmet.ac.uk)

The referees have opted to remain anonymous.

Review Timeline:

Submission Date:	14-Nov-2025
Editorial Decision:	07-Jan-2026
Revision Received:	11-May-2026
Accepted:	15-May-2026

Senior Editor: Kim Barrett

Reviewing Editor: Ken O'Halloran

Transaction Report:

(Note: With the exception of the correction of typographical or spelling errors that could be a source of ambiguity, letters and reports are not edited. Depending on transfer agreements, referee reports obtained elsewhere may or may not be included in this compilation. Referee reports are anonymous unless the Referee chooses to sign their reports.)

Re: JP-RP-2025-290512 "The influence of metaboreflex activation on pulmonary pressure with combined chemoreflex activation in acute and chronic hypoxia" by Lauren E Maier, Elliott J Jenkins, Andrew JM Douglas, Jack S Talbot, Alexandra Mackenzie Williams, Jonathan P Moore, Craig Steinback, Liam Corr, Zoe H. Adams, Fabio Giuseppe Laginestra, Markus Amann, Philip N Ainslie, Travis D Gibbons, and Mike Stembridge

Dear Dr Stembridge,

Thank you for submitting your manuscript to The Journal of Physiology. It has been assessed by a Reviewing Editor and by 1 expert referee and we are pleased to tell you that it is potentially acceptable for publication following satisfactory major revision.

Please advise your co-authors of this decision as soon as possible.

The referee reports are copied at the end of this email.

Please address all the points raised and incorporate all requested revisions or explain in your Response to Referees why a change has not been made. We hope you will find the comments helpful and that you will be able to return your revised manuscript within 2 months. If you require an extension, please contact journal staff: jp@physoc.org. Please note that this letter does not constitute a guarantee for acceptance of your revised manuscript.

Your revised manuscript should be submitted online using the link in your Author Tasks: Link Not Available. This link is accessible via your account as Corresponding Author; it is not available to your co-authors. If this presents a problem, please contact journal staff (jp@physoc.org). Image files from the previous version are retained on the system. Please ensure you replace or remove any files that are being revised.

If you do not wish to submit a revised version of your manuscript, you must inform our journal staff (jp@physoc.org) or reply to this email to request withdrawal. Please note that a manuscript must be formally withdrawn from the peer review process at one journal before it may be submitted to another journal.

TRANSPARENT PEER REVIEW POLICY: To improve the transparency of its peer review process, The Journal of Physiology publishes online as supporting information the peer review history of all articles accepted for publication. Readers will have access to decision letters, including Editors' comments and referee reports, for each version of the manuscript, as well as any author responses to peer review comments. Referees can decide whether or not they wish to be named on the peer review history document.

ABSTRACT FIGURES: Authors are expected to use The Journal's premium BioRender account to create/redraw their Abstract Figures. Information on how to access this account is here: https://physoc.onlinelibrary.wiley.com/journal/14697793/biorender-access.

This will enable Authors to create and download high-resolution figures. If authors have used the free BioRender service, they can use the instructions provided in the link above to download a high-resolution version suitable for publication.

The link provided should only be used for the purposes of this submission. Authors will be charged for figures created on this account if they are not related to this manuscript submission.

LANGUAGE EDITING AND SUPPORT FOR PUBLICATION: If you would like help with English language editing, or other article preparation support, Wiley Editing Services offers expert help, including English Language Editing, as well as translation, manuscript formatting, and figure formatting at www.wileyauthors.com/eeo/preparation. You can also find resources for Preparing Your Article for general guidance about writing and preparing your manuscript at www.wileyauthors.com/eeo/prepresources.

RIGOUR AND REPRODUCIBILITY: We recommend authors consult The Journal's Rigour and Reproducibility requirements to ensure that their manuscript is suitable for publication: https://physoc.onlinelibrary.wiley.com/pb-assets/hub-assets/physoc/documents/TJP-Rigour-and-Reproducibility-Requirements.pdf

REVISION CHECKLIST:

Check that your Methods section conforms to journal policy: https://jp.msubmit.net/cgi-bin/main.plex?form_type=display_requirements#methods.

Check that data presented conforms to the statistics policy: https://jp.msubmit.net/cgi-bin/main.plex?form_type=display_requirements#statistics.

Upload a full Response to Referees file. To create your 'Response to Referees': copy all the reports, including any comments from the Senior and Reviewing Editors, into a Microsoft Word, or similar, file and respond to each point, using font or background colour to distinguish comments and responses and upload as the required file type.

Please upload two versions of your manuscript text: one with all relevant changes highlighted and one clean version with no changes tracked. The manuscript file should include all tables and figure legends, but each figure/graph should be uploaded as separate, high-resolution files.

You may also upload:

- 'Potential Cover Art' for consideration as the issue's cover image
- Appropriate Supporting Information (Video, audio or data set: see https://jp.msubmit.net/cgi-bin/main.plex?form_type=display_requirements#supp).

We look forward to receiving your revised submission.

If you have any queries, please reply to this email and we will be pleased to advise.

Yours sincerely,

Kim Barrett
Senior Editor
The Journal of Physiology

REQUIRED ITEMS

- Author photo and profile. First or joint first authors are asked to provide a short biography (no more than 100 words for one author or 150 words in total for joint first authors) and a portrait photograph. These should be uploaded and clearly labelled together in a Word document with the revised version of the manuscript. See Information for Authors for further details.

- Please upload separate high-quality figure files via the submission form.

- Please include an Abstract Figure file and an Abstract Figure legend. An appropriate figure legend, which should not exceed 150 words in length, should be included in the main manuscript file. The Abstract Figure is a piece of artwork designed to give readers an immediate understanding of the research and should summarise the main conclusions. If possible, the image should be easily 'readable' from left to right or top to bottom. It should show the physiological relevance of the manuscript so readers can assess the importance and content of its findings. Abstract Figures should not merely recapitulate other figures in the manuscript. Please try to keep the diagram as simple as possible and without superfluous information that may distract from the main conclusion(s). Abstract Figures must be provided by authors no later than the revised manuscript stage and should be uploaded as a separate file during online submission labelled as File Type 'Abstract Figure'. Please also ensure that you include the figure legend in the main article file. All Abstract Figures should be created using BioRender. Authors should use The Journal's premium BioRender account to export high-resolution images. Details on how to use and access the premium account are included as part of this email.

EDITOR COMMENTS

Reviewing Editor:

Thank you for submitting your manuscript to The Journal of Physiology. An expert in the field has provided a thorough, fair-spirited critique, and I concur with the central message of the constructive comments provided. The study is well executed and the logistical challenges of field (and lab!) studies of this nature are acknowledged. The manuscript would benefit from a re-framing of the rationale for the study so that it is not viewed as esoteric, and rather seeks to provide fundamental knowledge advancing physiological understanding, perhaps with wider application. The referee has expressed concern that the manuscript in its present guise appears modestly incremental in its value. A final judgement in these terms would preclude acceptance for publication, but I do recognise that it is within the authors' gift to address this perceived weakness.

I acknowledge the referee's point of concern regarding sex as a variable of interest (an by extension whether there are sex differences which are not addressed in the current manuscript), but I recognise too the challenges for investigators in field studies where sample sizes are often restricted. I commend the authors for the mixed sex approach (addressing the issue of generalisability of findings) and I empathise with authors when a concerned referee or reader (rightly) raises the issue of

whether or not sex differences exist, whether they can be determined post-hoc and the conundrum of commenting even if underpowered to make comparisons (which is fraught with its own issues). I encourage you to engage in a fair-spirited way with the referee's interest in the matter (and the physiological relevance given reported sex differences in autonomic control)). It may be that it cannot adequately be addressed in the main manuscript, but I would encourage thorough commentary in the rebuttal as peer-review comments are published and serve as an additional resource to readers, providing in effect, a supplementary space to complement the manuscript, should it be accepted for publication. Of course, if robust comments and conclusions can be drawn from a re-evaluation of the data these should be incorporated into the main manuscript. If unclear, I agree with the referee that this limitation should be acknowledged.

I do wish to emphasize again that a major revision of the introduction is required to better frame the study.

We look forward to receiving a revised manuscript.

REFEREE COMMENTS

Referee #1:

This manuscript investigates how systolic pulmonary arterial pressure (PASP) responds to isolated and combined activation of the metaboreflex and peripheral chemoreflex during acute and chronic hypoxia. Using handgrip exercise and post-exercise circulatory occlusion under normoxic and isocapnic hypoxic conditions, the authors show that both reflexes independently elevate PASP, but their combined activation produces a strictly additive response, with no evidence of synergistic amplification. This pattern was consistent after short-term high-altitude acclimatization, despite clear ventilatory adaptations and higher baseline PASP. Overall, the study suggests that, unlike systemic cardiovascular variables, pulmonary vascular pressure integrates multiple autonomic inputs in an additive manner within the specific conditions tested. While the experimental design is well controlled and the topic is of physiological interest, several major issues limit the conceptual clarity, mechanistic framing, and interpretability of the findings.

- The introduction would benefit from substantial restructuring. Although it provides a broad overview of sympathetic and reflex-mediated responses across various physiological systems, the narrative does not converge on a clear physiological rationale for examining systolic pulmonary pressure (PASP) under combined metaboreflex and chemoreflex activation. The authors present several disparate examples of hyperadditive behaviour in systemic variables (e.g., heart rate, ventilation, arterial pressure) and imply that similar interactions might occur in the pulmonary circulation; however, the manuscript does not explain why such behaviour would be expected, or even mechanistically plausible, specifically for PASP. As a result, the rationale for the study remains underdeveloped. Further, the introduction does not clarify what makes the pulmonary vasculature uniquely relevant in this context, why its integrative reflex control might differ from that of the systemic circulation, or how identifying an additive versus divergent response would advance physiological understanding. Without a clear conceptual gap or a justified physiological prediction, the literature review feels fragmented and largely descriptive, rather than guiding the reader toward a coherent and compelling rationale for the experimental question. A more targeted and physiologically grounded rationale is needed to position the study within a meaningful mechanistic framework.

- The absence of any sex-based analysis represents a significant limitation. Although the sample size is modest, the study includes both men and women, and there is substantial evidence that sex influences autonomic regulation. Without even a descriptive comparison or acknowledgment of potential sex-specific differences, it is difficult to determine whether the observed PASP responses are consistent across sexes or disproportionately driven by one subgroup. At minimum, the manuscript should acknowledge this limitation and discuss its implications for interpretation, given the well-documented sex differences in reflex control.

- While the data clearly show that the net change in PASP during combined metaboreflex and chemoreflex activation is compatible with an additive response under the present experimental conditions, I would encourage the authors to moderate the breadth of some interpretative statements. The absence of a hyperadditive effect in this specific paradigm should not be taken to suggest that the integrative control of the pulmonary circulation is necessarily straightforward or governed by simple summation of inputs. Multiple excitatory drives may converge on the pulmonary vasculature and still exhibit non-linear

behaviour, context-dependent modulation, differential weighting, or ceiling effects that cannot be identified by comparing only two levels of activation. Likewise, if one of these reflex inputs were attenuated or selectively removed, the resulting change in PASP would not, a priori, be expected to scale proportionally. For these reasons, I recommend softening the language that implies broader mechanistic generalization and framing the conclusion more narrowly...e.g., noting that within the conditions tested, PASP responses were consistent with an additive summation of metaboreflex and chemoreflex influences.

END OF COMMENTS

EDITOR COMMENTS

Reviewing Editor:

Thank you for submitting your manuscript to The Journal of Physiology. An expert in the field has provided a thorough, fair-spirited critique, and I concur with the central message of the constructive comments provided. The study is well executed and the logistical challenges of field (and lab!) studies of this nature are acknowledged. The manuscript would benefit from a re-framing of the rationale for the study so that it is not viewed as esoteric, and rather seeks to provide fundamental knowledge advancing physiological understanding, perhaps with wider application. The referee has expressed concern that the manuscript in its present guise appears modestly incremental in its value. A final judgement in these terms would preclude acceptance for publication, but I do recognise that it is within the authors' gift to address this perceived weakness.

I acknowledge the referee's point of concern regarding sex as a variable of interest (an by extension whether there are sex differences which are not addressed in the current manuscript), but I recognise too the challenges for investigators in field studies where sample sizes are often restricted. I commend the authors for the mixed sex approach (addressing the issue of generalisability of findings) and I empathise with authors when a concerned referee or reader (rightly) raises the issue of whether or not sex differences exist, whether they can be determined post-hoc and the conundrum of commenting even if underpowered to make comparisons (which is fraught with its own issues). I encourage you to engage in a fair-spirited way with the referee's interest in the matter (and the physiological relevance given reported sex differences in autonomic control)). It may be that it cannot adequately be addressed in the main manuscript, but I would encourage thorough commentary in the rebuttal as peer-review comments are published and serve as an additional resource to readers, providing in effect, a supplementary space to complement the manuscript, should it be accepted for publication. Of course, if robust comments and conclusions can be drawn from a re-evaluation of the data these should be incorporated into the main manuscript. If unclear, I agree with the referee that this limitation should be acknowledged.

I do wish to emphasize again that a major revision of the introduction is required to better frame the study.

We look forward to receiving a revised manuscript.

Thank you for the thoughtful consideration given to our manuscript and the expert review provided. We concede that the original framing of the study did not generate a strong rationale for the research question. We have entirely redrafted this section, and now believe the physiological rationale and relevance are much clearer.

As a research team, we too are interested in the effects of biological sex as a variable. We chose not to test for sex differences as it was not the research question we set out to address. However, we acknowledge that there is evidence for sex-specific effects to both hypoxia and post exercise circulatory occlusion, and our sample would allow for exploratory investigation. Therefore, we have now tested for sex differences in the pulmonary pressure response and included the data in the manuscript. Further details are presented below.

Thank you for the continued consideration of our manuscript, which we believe is much improved following the peer-review process.

REFEREE COMMENTS

Referee #1:

This manuscript investigates how systolic pulmonary arterial pressure (PASP) responds to isolated and combined activation of the metaboreflex and peripheral chemoreflex during acute and chronic hypoxia. Using handgrip exercise and post-exercise circulatory occlusion under normoxic and isocapnic hypoxic conditions, the authors show that both reflexes independently elevate PASP, but their combined activation produces a strictly additive response, with no evidence of synergistic amplification. This pattern was consistent after short-term high-altitude acclimatization, despite clear ventilatory adaptations and higher baseline PASP. Overall, the study suggests that, unlike systemic cardiovascular variables, pulmonary vascular pressure integrates multiple autonomic inputs in an additive manner within the specific conditions tested. While the experimental design is well controlled and the topic is of physiological interest, several major issues limit the conceptual clarity, mechanistic framing, and interpretability of the findings.

Thank you for your detailed feedback and the constructive tone of your review. Please see below where we detail how we have addressed each of your points.

- The introduction would benefit from substantial restructuring. Although it provides a broad overview of sympathetic and reflex-mediated responses across various physiological systems, the narrative does not converge on a clear physiological rationale for examining systolic pulmonary pressure (PASP) under combined metaboreflex and chemoreflex activation. The authors present several disparate examples of hyperadditive behaviour in systemic variables (e.g., heart rate, ventilation, arterial pressure) and imply that similar interactions might occur in the pulmonary circulation; however, the manuscript does not explain why such behaviour would be expected, or even mechanistically plausible, specifically for PASP. As a result, the rationale for the study remains underdeveloped. Further, the introduction does not clarify what makes the pulmonary vasculature uniquely relevant in this context, why its integrative reflex control might differ from that of the systemic circulation, or how identifying an additive versus divergent response would advance physiological understanding. Without a clear conceptual gap or a justified physiological prediction, the literature review feels fragmented and largely descriptive, rather than guiding the reader toward a coherent and compelling rationale for the experimental question. A more targeted and physiologically grounded rationale is needed to position the study within a meaningful mechanistic framework.

We acknowledge the lack of conceptual clarity, and have taken the reviewer's comments onboard. The introduction has been completely redrafted, with specific thought given to the physiological rationale, the differences between the systemic and pulmonary vasculature, and the relevance of the knowledge generated. We hope the reviewer agrees that the introduction is vastly improved, and we are open to further changes if required.

- The absence of any sex-based analysis represents a significant limitation. Although the sample size is modest, the study includes both men and women, and there is substantial evidence that sex influences autonomic regulation. Without even a descriptive comparison or acknowledgment of potential sex-specific differences, it is difficult to determine whether the observed PASP responses are consistent across sexes or disproportionately driven by one subgroup. At minimum, the manuscript should acknowledge this limitation and discuss its implications for interpretation, given the well-documented sex differences in reflex control.

We appreciate the reviewer's concern to explore sex-based differences and agree there may be differences in the pulmonary artery pressure response (PASP) response to metaboreflex and/or chemoreflex activation between males and females (Fatemian *et al.,* 2016). As a research team we have an interest in studying sex differences (Williams *et al.*, 2022; Douglas *et al.*, 2024; Maier *et al.*, 2026). However, we did not explore sex differences in the current study for the issues raised by the Reviewing Editor e.g. mainly statistical power and the risk of a type 2 error.

However, we agree with the reviewer that this topic has sound physiological underpinning. Therefore, we have conducted *post-hoc* analyses on the change in PASP during hypoxia, PECO, and combined hypoxic PECO in both acute hypoxia and high-altitude conditions (i.e., Figures 2 and 4 of the manuscript). In study 1, there was no main effect of sex on ΔPASP ($p = 0.839$). Similarly, sex did not influence the ΔPASP response in study 2 with chronic hypoxia exposure ($p = 0.901$). We have not performed a post hoc power calculation given the inherent reliance on p values, but it appears highly unlikely there is a sex difference in the response. We have included these data in the results section of the revised manuscript, added a brief discussion of the lack of differences observed between sexes and acknowledged the small sample size in limitations. Details of these changes are pasted below.

Added to Results:

Study 1:

Sex-based comparisons

The change in PASP in females during isocapnic hypoxia (3.3 ± 2.3 mmHg), PECO (6.7 ± 7.4 mmHg) and combined isocapnic hypoxia and PECO (8.1 ± 4.4 mmHg) was similar compared to males during isocapnic hypoxia (4.5 ± 1.7 mmHg), PECO (4.2 ± 2.7 mmHg) and combined isocapnic hypoxia and PECO (8.6 ± 3.3 mmHg; main effect of sex, p = 0.839). Thus, participants were grouped together for analysis.

Study 2:

Sex-based comparisons

The change in PASP in females with high altitude (3.3 ± 4.8 mmHg), PECO (3.3 ± 2.5 mmHg) and combined high altitude and PECO (6.7 ± 6.5 mmHg) was similar compared to males at high altitude (4.1 ± 5.5 mmHg), PECO (5.0 ± 3.8 mmHg) and combined high altitude and PECO (9.2 ± 8.4 mmHg; main effect of sex, p = 0.901). Thus, participants were grouped together for analysis.

Added to Discussion:

Potential influence of sex

Finally, it is important to consider the role of sex in pulmonary vascular responses to chemo- and metaboreflex activation. Females may have a blunted early acclimatory response of pulmonary arterial systolic pressure to isocapnic hypoxia (Fatemian *et al.*, 2016)*, and cardiac responses may differ between sexes during concomitant chemo- and metaboreceptor activation (Boulet et al., 2022). However, post-hoc analyses did not identify an effect of sex on our a priori outcome; thus, sex does not appear to influence our interpretation.*

Added to Limitations:

The a priori question of this study was to determine whether there was a hyper-additive effect of the metabo- and chemoreflexes on PASP; thus, we may not have been powered to detect sex-based differences. Further, we were unable to control for menstrual cycle phase or contraceptive use in study 2, due to the nature of research expeditions, and chose not to control for these in study 1, as it was not the a priori research question. As a result, the role of sex in these outcomes is further confounded by variable hormone concentrations.

We have also graphed our data by sex and included here so they can form part of the record of peer review, but chose not to include the figures in the manuscript as we fear this would complicate the results section.

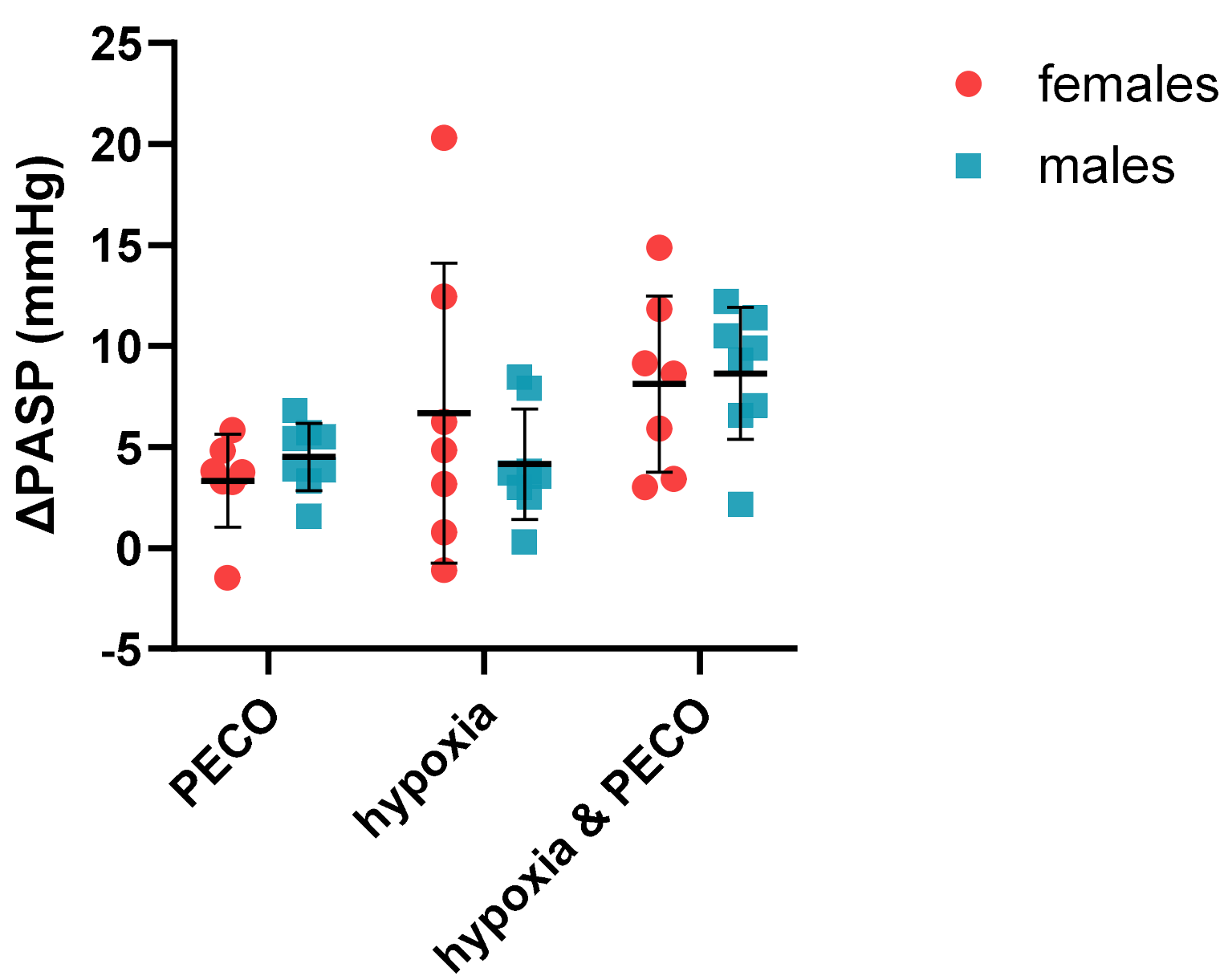

Fig S1. The change in PASP with PECO, acute isocapnic hypoxia, and combined PECO and isocapnic hypoxia. There was no main effect of sex ($p = 0.839$).

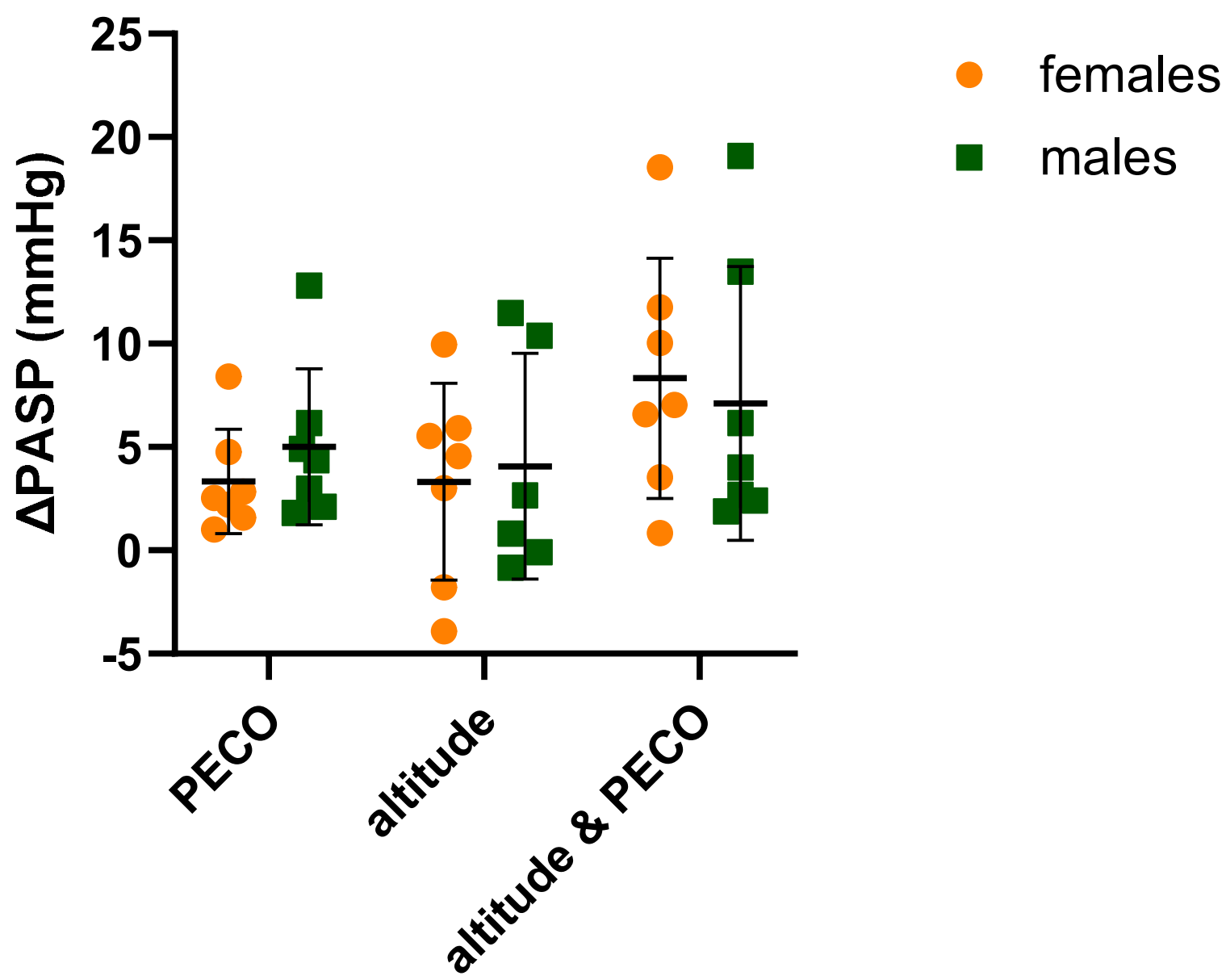

Fig S2. The change in PASP with PECO, altitude, and combined PECO and altitude. There was no main effect of sex ($p = 0.901$).

- While the data clearly show that the net change in PASP during combined metaboreflex and chemoreflex activation is compatible with an additive response under the present experimental conditions, I would encourage the authors to moderate the breadth of some interpretative statements. The absence of a hyperadditive effect in this specific paradigm should not be taken to suggest that the integrative control of the pulmonary circulation is necessarily straightforward or governed by simple summation of inputs. Multiple excitatory drives may converge on the pulmonary vasculature and still exhibit non-linear behaviour, context-dependent modulation, differential weighting, or ceiling effects that cannot be identified by comparing only two levels of activation. Likewise, if one of these reflex inputs were attenuated or selectively removed, the resulting change in PASP would not, a priori, be expected to scale proportionally. For these reasons, I recommend softening the language that implies broader mechanistic generalization and framing the conclusion more narrowly...e.g., noting that within the conditions tested, PASP responses were consistent with an additive summation of metaboreflex and chemoreflex influences.

We thank the review for reminding us of the context-specific nature of our results. We did not intend to over-generalise but acknowledge that our language did not reflect this. We have amended the manuscript to tone down our assertions in line with the reviewer's suggestion, particularly around our summarising statements, conclusions and in our limitations.

References

Douglas AJM, Talbot JS, Perkins D, Dawkins TG, Oliver JL, Lloyd RS, Ainslie PN, McManus A, Pugh CJA, Lord RN & Stembridge M. (2024). The influence of maturation and sex on intracranial blood velocities during exercise in children. *J Appl Physiol (1985)* **136,** 451-459.

Fatemian M, Herigstad M, Croft QP, Formenti F, Cardenas R, Wheeler C, Smith TG, Friedmannova M, Dorrington KL & Robbins PA. (2016). Determinants of ventilation and pulmonary artery pressure during early acclimatization to hypoxia in humans. *The Journal of physiology* **594,** 1197-1213.

Maier LE, Vanden Berg ER, Simpson L, Ewalts M, Foster K, Baylis J, Gasho C, Macleod D, van Diepen S, Anholm J, Lawley J, Ainslie PN, Gibbons TD, Stembridge M, Moore J & Steinback CD. (2026). The effect of sex on the isolated and combined α- and β-adrenergic control of blood flow during handgrip in adults at high altitude: An exploratory study. *Physiol Rep* **14,** e70754.

Williams AM, Levine BD & Stembridge M. (2022). A change of heart: Mechanisms of cardiac adaptation to acute and chronic hypoxia. *The Journal of Physiology* **600,** 4089-4104.

Dear Mike,

Re: JP-RP-2026-290512R1 "The influence of metaboreflex activation on pulmonary pressure with combined chemoreflex activation in acute and chronic hypoxia" by Lauren E Maier, Elliott J Jenkins, Andrew JM Douglas, Jack S Talbot, Alexandra Mackenzie Williams, Jonathan P Moore, Craig Steinback, Liam Corr, Zoe H. Adams, Fabio Giuseppe Laginestra, Markus Amann, Philip N Ainslie, Travis D Gibbons, and Mike Stembridge

We are pleased to tell you that your paper has been accepted for publication in The Journal of Physiology.

The last Word (or similar) version of the manuscript provided will be used by the Production Editor to prepare your proof. When this is ready you will receive an email containing a link to Wiley's Online Proofing System. The proof should be thoroughly checked and corrected as promptly as possible.

Authors should note that it is too late at this point to offer corrections prior to proofreading. Major corrections at proof stage, such as changes to figures, will be referred to the Editors for approval before they can be incorporated. Only minor changes, such as to style and consistency, should be made at proof stage. Changes that need to be made after proof stage will usually require a formal correction notice.

All queries at proof stage should be sent to: TJP@wiley.com.

Following copyediting and typesetting the accepted version will be published online.

Best wishes,

Kim Barrett
Senior Editor
The Journal of Physiology

IMPORTANT POINTS TO NOTE FOLLOWING ACCEPTANCE OF YOUR PAPER:

• IMPORTANT NOTICE ABOUT OPEN ACCESS: To assist authors whose funding agencies mandate immediate public access to published research findings, The Journal of Physiology allows authors to pay an Open Access (OA) fee to have their papers made freely available immediately on publication.

The Corresponding Author will receive an email from Wiley with details on how to register or log in to Wiley Authors where you will be able to place an order.

• You can check if your funder or institution has a Wiley Open Access Account here:
https://authors.wiley.com/author-resources/Journal-Authors/open-access/author-compliance-tool.html

• TRANSPARENT PEER REVIEW POLICY: To improve the transparency of its peer review process, The Journal of Physiology publishes online as supporting information the peer review history of all articles accepted for publication. Readers will have access to decision letters, including Editors' comments and referee reports, for each version of the manuscript as well as any author responses to peer review comments. Referees can decide whether or not they wish to be named on the peer review history document.

• You can help your research get the attention it deserves! Check out Wiley's free Promotion Guide for best-practice recommendations for promoting your work at: www.wileyauthors.com/eeo/guide. You can learn more about Wiley Editing Services which offers professional video, design, and writing services to create shareable video abstracts, infographics, conference posters, lay summaries, and research news stories for your research at: www.wileyauthors.com/eeo/promotion.

• If you would like to receive our 'Research Roundup', a monthly newsletter highlighting the cutting-edge research published in The Physiological Society's family of journals (The Journal of Physiology, Experimental Physiology, Physiological Reports, The Journal of Nutritional Physiology and The Journal of Precision Medicine: Health and Disease), please click this link, fill in your name and email address and select 'Research Roundup':
https://www.physoc.org/journals-and-media/membernews

EDITOR COMMENTS

Reviewing Editor:

Thank you for your responses to the points raised in the previous round of queries and for making comprehensive changes to the introduction. I am pleased to report that all points have been satisfactorily addressed. Thank you also for your positive comments regarding the peer review process and for engaging positively with same. The additional analyses provided in your responses are a useful complement to the main manuscript. Congratulations!

REFEREE COMMENTS

Referee #1:

No further comments.
